# Pirikool® 300 CS, a new long-lasting capsule suspension formulation of the organophosphate insecticide pirimiphos-methyl for indoor residual spraying against pyrethroid-resistant malaria vectors

**DOI:** 10.1371/journal.pone.0267229

**Published:** 2022-04-18

**Authors:** Augustin Fongnikin, Abibath Odjo, Joel Akpi, Laurette Kiki, Corine Ngufor

**Affiliations:** 1 Centre de Recherche Entomologique de Cotonou, Cotonou, Benin; 2 Pan African Malaria Vector Research Consortium (PAMVERC), Cotonou, Benin; 3 London School of Hygiene & Tropical Medicine, London, United Kingdom; National Institute for Communicable Diseases, SOUTH AFRICA

## Abstract

**Background:**

Indoor residual spraying (IRS) using a capsule suspension formulation of the organophosphate insecticide, pirimiphos-methyl, has provided substantial malaria control in many communities in Africa. However, only one brand of this product has been recommended by the World Health Organisation for IRS. To help increase the diversity of the portfolio of IRS insecticides and offer suitable options to procurers and malaria vector control programmes, additional product brands of this highly effective and long-lasting insecticide formulation for IRS will be needed.

**Methods:**

We evaluated the efficacy of Pirikool® 300CS, a new capsule suspension formulation of pirimiphos-methyl developed by Tianjin Yorkool, International Trading, Co., Ltd in standard WHO laboratory bioassays and experimental hut studies. The efficacy of the insecticide applied at 1000mg/m^2^ was assessed in laboratory bioassays for 6 months on cement, plywood and mud block substrates and for 12 months in cement and mud-walled experimental huts against wild free-flying pyrethroid-resistant *Anopheles gambiae* sensu lato in Covè, Benin. Actellic® 300CS, a WHO-recommended capsule suspension formulation of pirimiphos-methyl was also tested. WHO cylinder tests were performed to determine the frequency of insecticide resistance in the wild vector population during the hut trial.

**Results:**

The vector population at the hut station was resistant to pyrethroids but susceptible to pirimiphos-methyl. Overall mortality rates of wild free-flying pyrethroid-resistant *An*. *gambiae* (s.l.) entering Pirikool®300CS treated experimental huts during the 12-month trial were 86.7% in cement-walled huts and 88% in mud-walled huts. Mortality of susceptible *An*. *gambiae* (Kisumu) and pyrethroid-resistant *An*. *gambiae* s.l. (Covè) mosquitoes in monthly wall cone bioassays on Pirikool® 300CS treated hut walls remained over 80% for 10–12 months. The laboratory bioassays corroborated the hut findings with Pirikool® 300CS on mud and wood block substrates but not on cement block substrates.

**Conclusion:**

Indoor residual spraying with Pirikool® 300CS induced high and prolonged mortality of wild pyrethroid-resistant malaria vectors for 10–12 months. Addition of Pirikool®300CS to the current portfolio of IRS insecticides will provide an extra choice of microencapsulated pirimiphos-methyl for IRS.

## Introduction

Indoor residual spraying (IRS) is one of the principal methods used to control malaria on a large scale [1, 2]. Due to its ability to rapidly reduce vector populations and transmission, IRS has contributed significantly to the remarkable reductions in malaria burden observed in endemic countries [3]. Its efficacy depends largely on the characteristics of local vector populations, their continued susceptibility to the insecticides used, and its residual lifespan on treated home wall substrates.

Insecticides from four major classes of compounds are available for IRS against malaria vectors: pyrethroids, carbamates, organophosphates and more recently neonicotinoids [1]. The organochlorine DDT, though not prequalified, can be used for IRS only if no other equally effective and efficient alternative is available. A recent study on the trends of insecticides used for IRS demonstrated a remarkable shift from DDT and pyrethroids after 2010 to carbamates and organophosphates [4]. A wettable powder formulation of the carbamate bendiocarb and an emulsifiable concentrate formulation of the organophosphate pirimiphos-methyl were initially used by many IRS programmes [4, 5]. Though these formulations showed high toxicity against malaria vectors, their efficacy was short-lived (2–4 months) requiring multiple rounds of logistically challenging IRS campaigns to achieve effective malaria control in holo-endemic areas in Africa [6, 7]. This necessitated the development of longer-lasting formulations of these insecticides for use in areas with long transmission seasons [5]. A new microencapsulated formulation of pirimiphos-methyl (Actellic® 300CS) which demonstrated vastly improved residual duration up to 12 months after application in experimental hut trials in Benin and Tanzania [8, 9] was eventually recommended by WHO [10]. Largescale application of this formulation for IRS in sub-Saharan Africa [5] provided substantial control of mosquito vectors and malaria in many settings [11–18].

To help increase the availability of vector control products for use in endemic countries, a larger market with a wider variety of product formulations and brands needs to be available to procurers. The Vector Control Product Assessment Team of the WHO Pre-Qualification Unit (PQT/VCP) was recently established to increase access to safe, high-quality, effective vector control products through evaluations, inspections, and guidance [19]. While there are 19 pyrethroid insecticide products available for IRS on the PQT/VCP list of pre-qualified vector control products, only one product brand of the long-lasting microencapsulated formulation of pirimiphos-methyl for IRS is on the list to date [20]. Given the utility of this insecticide for IRS, new effective product options need to be developed to provide additional choices for IRS.

In the present study, we evaluated the efficacy of Pirikool® 300CS, a new microencapsulated formulation of pirimiphos-methyl developed by Tianjin Yorkool, International Trading, Co., Ltd in standard WHO phase I laboratory bioassays and phase 2 experimental hut studies [21]. The insecticide was tested for its efficacy and residual activity on various local wall substrates (cement, mud and wood) against laboratory maintained susceptible and resistant strains of *An*. *gambiae* s.l. and in an experimental hut trial against wild free-flying pyrethroid-resistant *An*. *gambiae* s.l. in southern Benin, West Africa. Actellic® 300CS,a WHO/PQ-listed micro-encapsulated organophosphate IRS insecticide, was also tested for comparison.

## Methods

### Phase I laboratory evaluation

#### Preparation and treatment of block substrates

Three major types of substrates used in housing construction in Benin (cement, mud and wood) were formed into circular blocks (9cm diameter and 5 cm thickness) and used for the laboratory bioassays. Cement blocks consisted of a mixture of commercial cement (Larfage, Benin) and sand at a 1:1 ratio while mud blocks were made by mixing mud paste with 10% cement in line with local practices. These blocks were preserved at 27±2°C and 80±10% RH for 30 days before insecticide application. Wooden blocks were simply cut out of locally available wooden planks. The substrates were treated using a potter tower sprayer to achieve a homogeneous and accurate deposit of the target concentration of active ingredients per unit area. To improve the quality of the treatment applications, each block was weighed before and after treatment to assess the quantity of insecticide solution applied; only blocks that received a volume within 20% range of the target were used for bioassays. All treated blocks were stored, unsealed at 30±2°C, 80±10% RH in between bioassays. Four replicate blocks of each substrate type were prepared for each of the following insecticides:

1. Pirikool® 300 CS (Tianjin Yorkool International Trading, Co., Ltd) a capsule suspension formulation of pirimiphos-methyl containing 300mg of active ingredient per litre and applied at 1000 mg/m^2^.
2. Actellic® 300 CS (Syngenta Crop Protection AG), a WHO/PQ listed capsule suspension formulation of pirimiphos-methyl also containing 300mg of active ingredient per litre and applied at 1000 mg/m^2^.
3. K-Othrin WG25 (Bayer CropScience), a WHO/PQ listed wettable granule formulation of deltamethrin for IRS applied at 25 mg/m^2^.

Four untreated blocks of each substrate type were also prepared and tested as controls alongside the treated blocks.

#### Residual efficacy on treated block substrates

The residual efficacy of the insecticides applied on the treated block substrates were assessed in monthly cone bioassays following WHO guidelines [20]. Two–Five (2–5) days old unfed laboratory-maintained mosquitoes of the susceptible *Anopheles gambiae* Kisumu strain (~80 mosquitoes per substrate per treatment) and the pyrethroid-resistant *Anopheles gambiae* s.l. Cové strain (~40 mosquitoes per substrate per treatment) were exposed for 30 min in replicates of 10 mosquitoes per block. Four blocks were tested per treatment and control. The laboratory cone bioassays were conducted one week after treatment and subsequently at monthly intervals for up to 6 months. Mortality was recorded 24 hours post-exposure.

### Experimental hut trial

#### Study site and experimental huts

The experimental hut study was performed at a Phase II experimental hut station in Covè, southern Benin (7°14’N 2°18’E) belonging to the CREC/LSHTM collaborative research programme. The field site is in an irrigated valley producing rice almost year-round which provides suitable breeding habitats for mosquitoes. The rainy season extends from March to October and the dry season from November to February. *Anopheles coluzzii* and *An*. *gambiae* sensu stricto (s.s.) occur in sympatry, with the latter present at lower densities and predominantly in the dry season. The vector population is susceptible to organophosphates and carbamates but exhibits intense resistance to pyrethroids (200-fold). Molecular genotyping and microarray studies have demonstrated a high frequency of the knockdown resistance L1014F allele (>90%) and overexpression of CYP6P3, an enzyme associated with pyrethroid detoxification [22]. The trial was carried out over 12 months between September 2019 and September 2020 in 9 experimental huts of West African design. The experimental huts are built on cement plinths surrounded by water-filled moats to prevent entry of scavenging ants and have veranda traps to capture exiting mosquitoes. The walls are made of brick plastered with either cement or mud on the inside, with a corrugated iron roof. The hut ceilings were covered with palm thatch. Each hut had window slits (1-cm gap) on each wall through which mosquitoes entered.

#### Susceptibility tests

To determine the frequency of insecticide resistance in the wild vector population during the hut trial, WHO cylinder bioassays were performed with 2–5-day old adult F1 female mosquitoes emerging from larvae collected from breeding sites close to the experimental huts. Approximately 100 female mosquitoes per insecticide were exposed for 1 h in batches of 25 to alpha-cypermethrin 0.05%, permethrin 0.75%, deltamethrin 0.05% and pirimiphos-methyl 0.25% treated filter papers. Knockdown was recorded after 1 h and mortality after a 24 h holding period.

#### Experimental hut treatments

The performance of Pirikool® 300CS was compared to the reference Actellic®300CS in the experimental hut trial. To prevent any contamination from previous trials, hut walls were refurbished by replastering with cement or mud and allowed to cure for 1 month prior to the evaluation. Two replicate huts were prepared per insecticide treatment. A total of nine treatments were evaluated in the experimental huts (Table 1).

**Table 1:** Experimental hut treatments.

Hut Number	Treatments	Wall Substrates	Replicate Number	IRS application dose (mg/m²)
**1**	Control (Untreated hut)	Cement	1	N/A
**2**	Actellic® 300CS	Cement	1	1000
**3**	Actellic® 300CS	Cement	2	1000
**4**	Actellic® 300CS	Mud	1	1000
**5**	Actellic® 300CS	Mud	2	1000
**6**	Pirikool® 300CS	Cement	1	1000
**7**	Pirikool® 300CS	Cement	2	1000
**8**	Pirikool® 300CS	Mud	1	1000
**9**	Pirikool® 300CS	Mud	2	1000

The walls and ceiling of each experimental hut was sprayed using a calibrated Hudson Xpert® compression sprayer equipped with an 8002 flat-fan nozzle. Prior to spraying, hut walls were measured, and spray swaths were pre-marked using chalk to improve the accuracy of spraying. Each swath was sprayed from the top to the bottom using the predetermined lance speed. Prior to spraying, the required quantity of Pirikool®300CS and Actellic®300CS was calculated and weighed. The Insecticide solutions were prepared by mixing with the required volumes of water. After spraying each hut, the insecticide solution left in the spray tank was measured to determine the volume sprayed. Spray volumes were within 30% of the target.

#### Assessing the quality of spray applications?

Before spraying, 5 filter papers (WHATMAN No.1) measuring 5cm x 5cm were fixed at 5 positions on the hut walls (1 per wall and one on the ceiling) to be sprayed. After spraying, they were left to dry for 1 hour and then wrapped in aluminium foil and stored at 4°C (+/-2°c) after which they were shipped within 2 weeks after IRS application to the International Institute of Biotechnology and Toxicology (IIBAT), India for chemical analysis.

#### Hut trial procedure

Nine consenting adult human volunteers slept in the huts from 20:00 to 05:00 each night of the study to attract mosquitoes into the hut and were rotated between huts on successive nights to adjust for any variation in individual attractiveness to mosquitoes using a Latin Square Design. In the morning of each day of the trial, mosquitoes were collected from the room and veranda trap of each hut and placed in correspondingly labelled plastic cups using a torch and aspirator. All mosquitoes (live and dead) found in the veranda trap were collected while only dead mosquitoes were collected from inside the room of the experimental hut. Live mosquitoes in the room were allowed until the evening to interact with the treatment and leave the room into the veranda trap. All mosquitoes remaining in the room and veranda were collected in the evening before the sleepers entering the huts. The mosquitoes were transferred to the laboratory for morphological identification and scoring of blood-feeding and mortality. Live mosquitoes were held in cups with 10% glucose solution to assess delayed mortality 24 hours post-exposure.

#### Outcome measures of experimental hut trial

The following outcome measures were used to assess the efficacy of the treatments in the experimental huts:

- Exiting rates–the proportion of mosquitoes found in the exit traps compared with the total number found in the hut and traps.
- Blood feeding rate–the proportion of blood-fed mosquitoes collected in the experimental hut.
- Mortality–the proportion of mosquitoes found dead in the hut after 24 hours.

#### Monthly residual bioassays on treated hut walls

To assess the residual efficacy of the insecticides on different experimental hut wall substrates (mud and cement), monthly cone bioassays were performed using pyrethroid-resistant (Covè) and insecticide-susceptible (Kisumu) strains of *An*. *gambiae* s.l. In each hut, a total of 5 cones were fixed to each treated surface (1 per wall + 1 on the ceiling). Fifty (50) unfed mosquitoes (2–5 days old) mosquitoes of each strain were exposed for 30 minutes to each hut treatment in batches of 10 mosquitoes per cone according to WHO IRS guidelines [17]. Knockdown was recorded after 60 minutes and mortality after a 24-hour holding period.

### Ethical considerations

Ethical approval for the study was obtained from the Ethics Review Committee of the Ministry of Health Benin (CNERS No. 39). All human volunteer sleepers gave written informed consent prior to their participation; where necessary, the consent form and information sheet were explained in their local language. They were offered a free course of chemoprophylaxis prior to their participation in the study. A stand-by nurse was available for the duration of the trial to assess any cases of fever or adverse reactions to test items and any confirmed cases of malaria were treated free of charge at a local health facility.

### Statistical analysis

Each proportional experimental hut trial outcome (blood-feeding, exophily and mortality) was analysed using logistic regression analysis on Stata version 15.1 with adjustments for repeated measures and random effects of sleepers and huts replicates.

## Results

### Laboratory cone bioassay results

**Fig 1:**
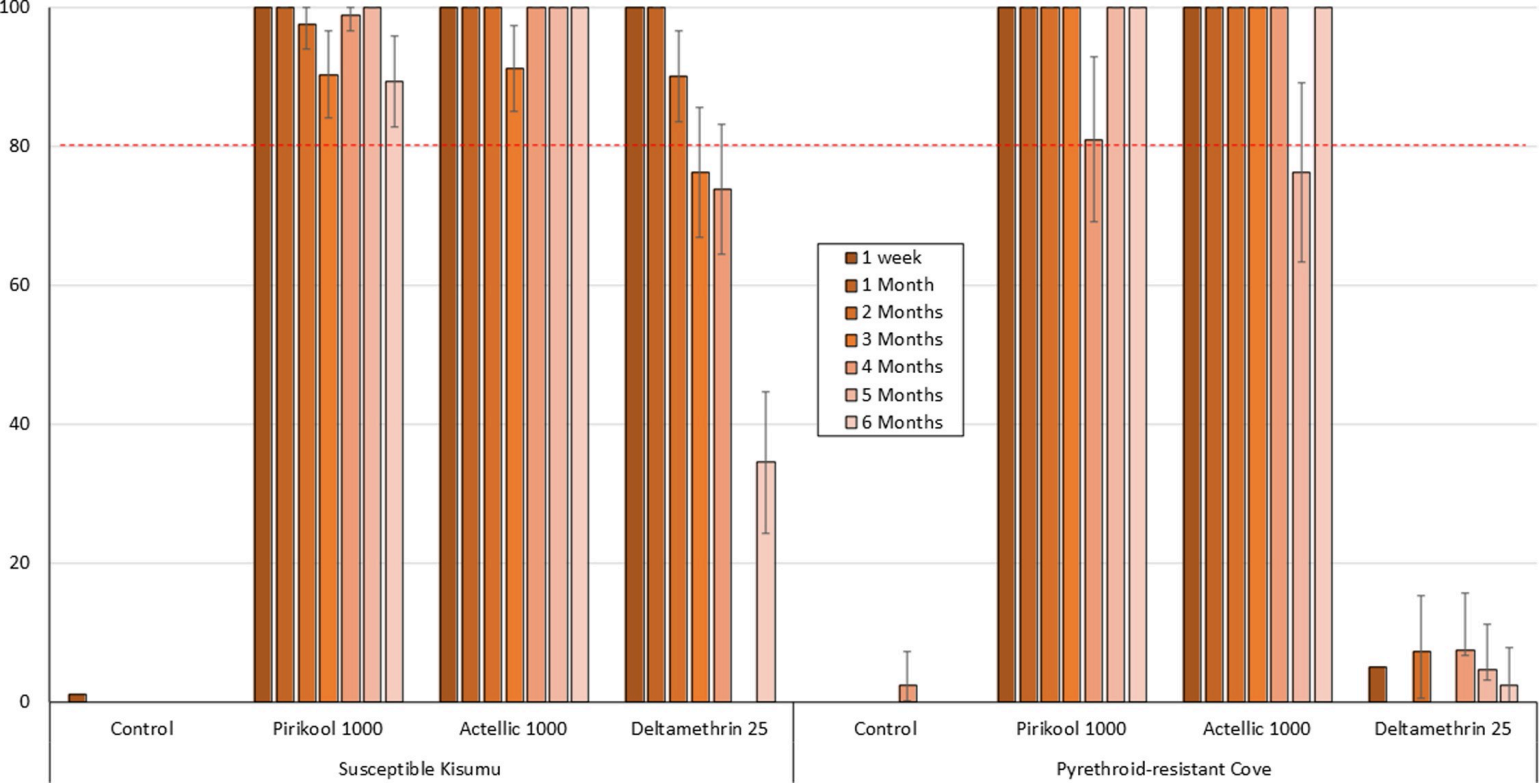
% Mortality (24h) of susceptible *An gambiae* Kisumu and pyrethroid-resistant *An gambiae* s.l. Cove strains in monthly laboratory cone bioassays on **Mud** block substrates. ~40 mosquitoes of each strain were tested on each treatment per time point.

**Fig 2:**
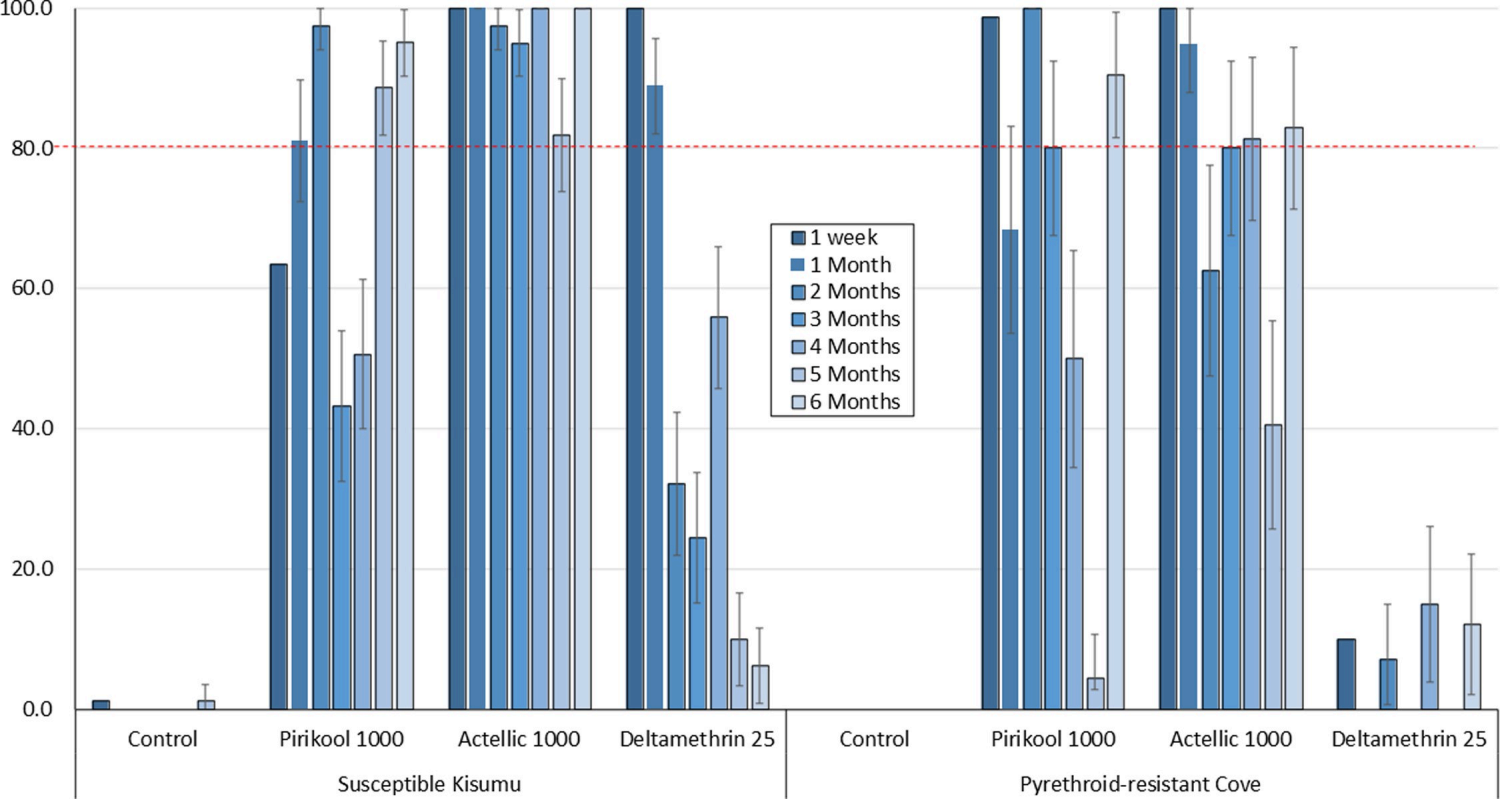
% Mortality (24h) of susceptible *An gambiae* Kisumu and pyrethroid-resistant *An gambiae* s.l. Cove strains in monthly laboratory cone bioassays on **Cement** block substrates. ~40 mosquitoes of each strain were tested on each treatment per time point.

**Fig 3:**
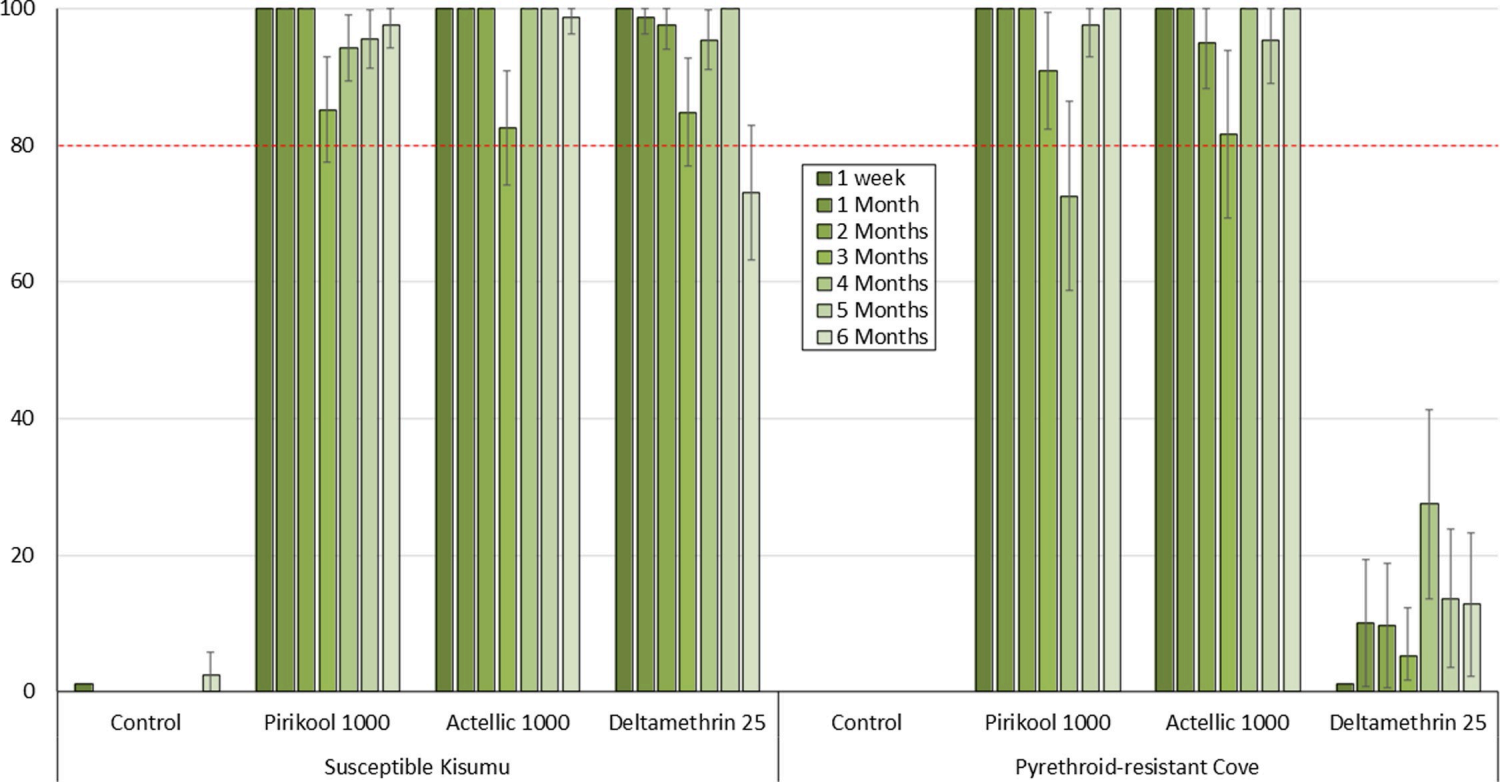
% Mortality (24h) of susceptible *An gambiae* Kisumu and pyrethroid-resistant *An gambiae* s.l. Cove strains in monthly laboratory cone bioassays on **Plywood** block substrates. ~40 mosquitoes of each strain were tested on each treatment per time point.

The monthly cone bioassay results are presented in Figs 1–3 for mortality rates for both susceptible *An gambiae* Kisumu and pyrethroid-resistant Cove mosquitoes exposed to the mud, cement and wood block substrates respectively.

#### Mud substrates

Mortality rates with both Pirikool®300CS and Actellic®300CS on mud substrates were >80% for all 6 months post-treatment with both susceptible Kisumu and pyrethroid-resistant Cove strains (Fig 1). The performance of Pirikool®300CS was therefore comparable to Actellic®300CS on mud substrates with both strains. Mortality with deltamethrin was very high with the pyrethroid susceptible Kisumu strain for the first 2 months but remained <10% with pyrethroid-resistant Cove throughout the study.

#### Cement substrates

Mortality rates with Pirikool® 300CS on cement substrates were lower than what was observed with Actellic® 300CS, especially for the susceptible Kisumu strain (Fig 2). Actellic® 300CS killed >80% of susceptible Kisumu for 6 months (Fig 2). With the pyrethroid-resistant, Cove strain mortality with both products was very variable. With the susceptible Kisumu strain, Actellic® 300CS outperformed Pirikool® 300CS but did not differ too much in performance against the pyrethroid-resistant Cove strain. The performance of Pirikool® 300CS on cement blocks was lower than what was observed with mud blocks probably due to a difference in surface texture. Mortality with deltamethrin was >80% for only 1 month with the susceptible Kisumu strain and <20% with the Cove strain throughout the study.

#### Plywood substrates

Mortality rates with Pirikool® 300CS on plywood substrates were generally similar to what was observed with Actellic® 300CS (Fig 3). Both products killed >80% of susceptible Kisumu for all 6 months of the study. There was a slight decrease in mortality with Pirikool® 300CS against the pyrethroid-resistant Cove strain at 4 months, but this quickly picked up and remained >80% for the rest of the study. Mortality with deltamethrin was very high with the pyrethroid susceptible Kisumu strain for the first 5 months but remained <30% with pyrethroid-resistant Cove throughout the study.

### Experimental hut results

#### WHO resistance bioassays

Mortality in WHO susceptibility cylinder bioassays was <10% for alpha-cypermethrin, permethrin and deltamethrin thus confirming the high levels of pyrethroid resistance in the Covè vector population. Mortality with pirimiphos-methyl 0.25% treated papers was 100%, demonstrating susceptibility to organophosphates (Table 2).

**Table 2:** Mortality of wild pyrethroid-resistant *Anopheles gambiae* s.l. from Covè in WHO susceptibility cylinder bioassays. ~100 mosquitoes were exposed to each insecticide in batches of 25 per cylinder.

Insecticide	Total exposed	Total dead	% dead
Pirimiphos-methyl 0.25%	100	100	100
Alpha-cypermethrin 0.05%	100	9	9
Permethrin 0.75%	97	8	8.2
Deltamethrin 0.05	98	5	5.1
Control	100	0	0

#### Mosquito entry and exiting rates

The experimental hut results are summarised in Table 3. A total of 28, 635 wild female *An*. *gambiae* s.l. were collected in the experimental huts during the trial at Covè. A significantly higher number of mosquitoes entered the control hut compared to the treated huts (Table 3). The proportion exiting the control hut was 40.6%. With all hut wall substrates, exiting rates with Actellic® 300CS (56.2%–75.9%) and the Pirikool® 300CS (51.6%–58%) were significantly higher than the control hut (40.6%, P<0.05). More detailed results from the hut trial are provided in S1 Table.

**Table 3:** Results with wild free-flying pyrethroid-resistant *An*. *gambiae* s.l. mosquitoes in experimental huts in Cove, Benin.

Hut treatments	Control	Actellic Cement		Actellic Mud		Pirikool Cement		Pirikool Mud	
Replicate	-	1	2	1	2	1	2	1	2
Total females caught	5340	2228	2500	2557	2966	3591	2497	3359	3597
N exiting	2 170	1 692	1 508	1 437	1 956	1 873	1 318	1 733	2 086
% Exiting	40.6	75.9	60.3	56.2	65.9	52.2	52.8	51.6	58.0
N blood-fed	5 228	2 161	2 423	2 486	2 869	3 486	2 432	3 241	3 505
% Blood fed	97.9	97.0	96.9	97.2	96.7	97.1	97.4	96.5	97.4
N dead	46	1 184	1 794	1 896	1 954	3 110	2 166	2 960	3 142
% Mortality	0.9	53.1	71.8	74.1	65.9	86.6	86.7	88.1	87.4
95% Conf Interval	(0.6–1.1)	(51–55)	(70–73)	(72–76)	(64–68)	(85–88)	(85–88)	(87–89)	(86–88)

#### Blood feeding rates

As expected of IRS treatments, blood-feeding rates were generally very high across all treatments (ranging between 96 and 98%) and did not show any difference relative to the control (98%) (Table 3). No significant difference in blood-feeding rates was also detected between treated huts for any of the wall substrates.

#### Mortality rates over 12 months

**Fig 4:**
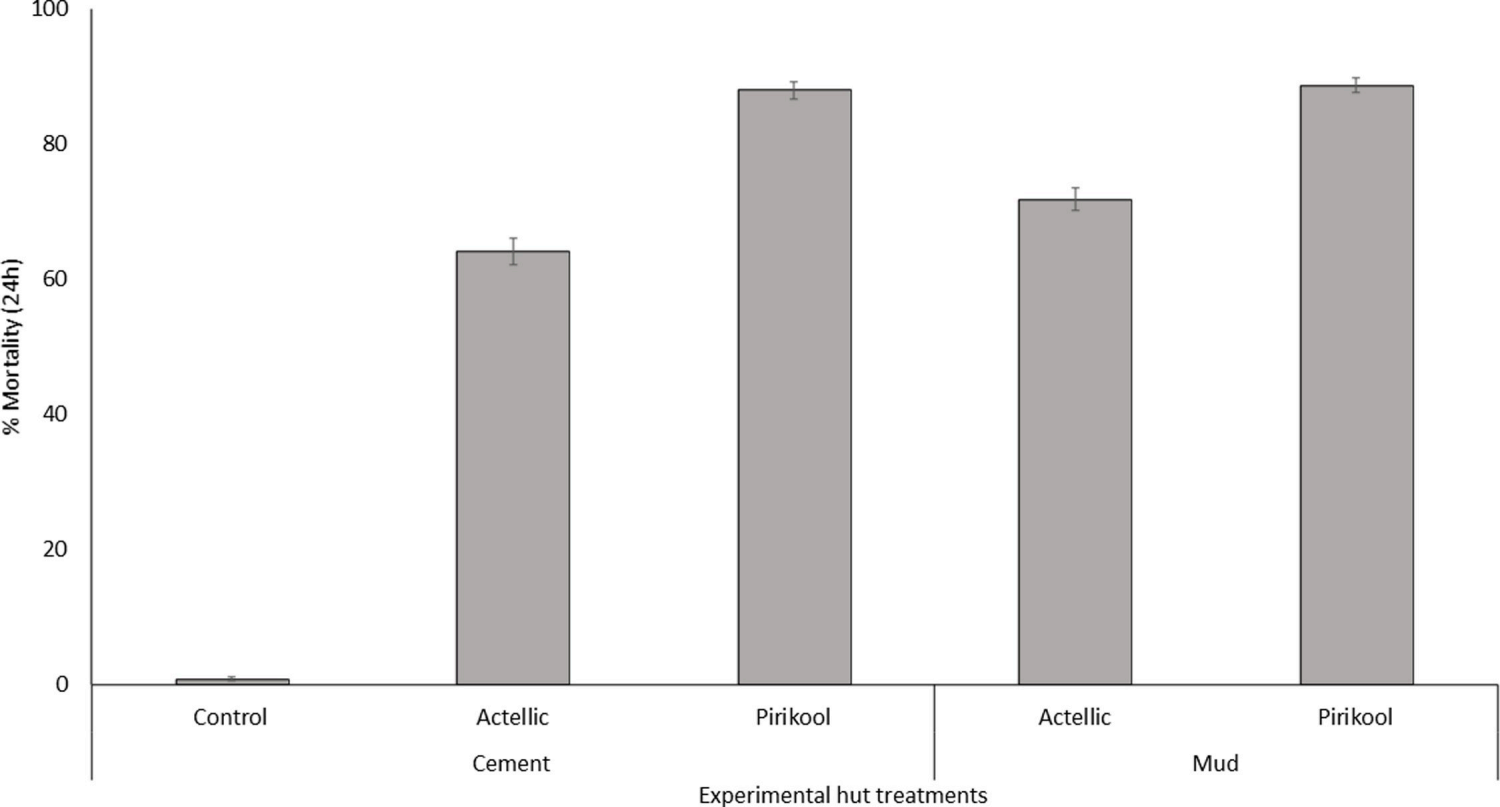
Overall mortality of wild free-flying pyrethroid-resistant *Anopheles gambiae* s.l in cement and mud-walled experimental huts in Cove, Benin. Each bar represents overall % mortality (24h) for each insecticide treatment on each wall substrate over 12 months. Data for replicate huts are pooled together to provide single estimates at each time point per treatment. Error bars represent 95% confidence intervals.

The overall mortality rates recorded after 24 hours over the 12-month trial and pooled for each treatment are presented in Fig 4. Mortality of wild pyrethroid-resistant *An*. *gambiae* s.l. in the control untreated hut was 0.9%. Mortality was 86.7–88% with Pirikool®300CS treated huts and 66.5%-70% with Actellic® 300CS. For each insecticide type, no difference in mortality was observed between the substrate types for each insecticide (p>0.05).

### Residual efficacy against free flying *Anopheles gambiae* s.l.

**Fig 5:**
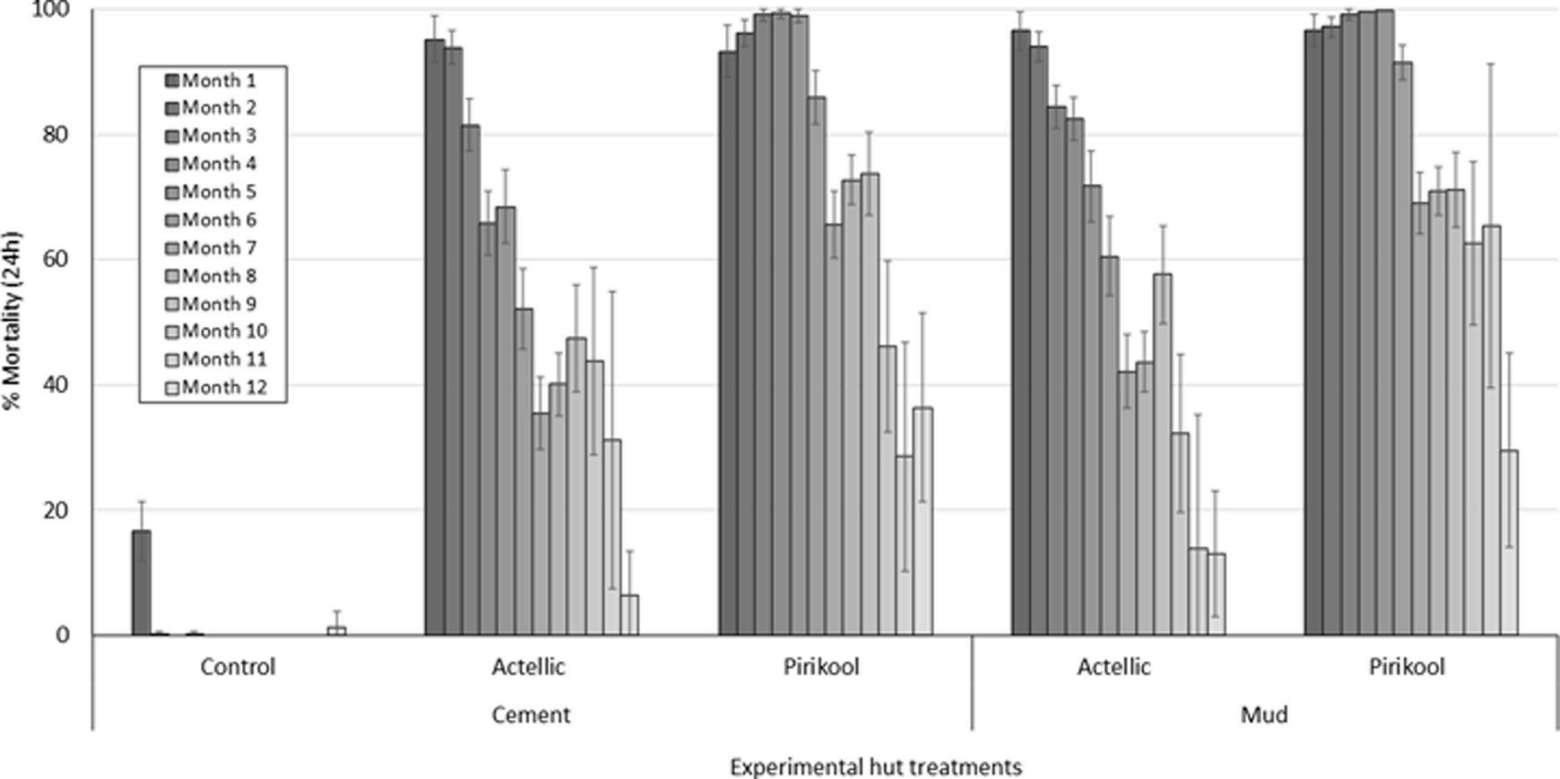
Monthly mortality rates of wild free-flying pyrethroid-resistant *An*. *gambiae* s.l. entering IRS treated cement and mud-walled experimental huts in Cove, Benin. Each bar represents % mortality (24h) over each successive month of the trial for each treatment. Data for replicate huts are pooled together to provide single estimates at each time point per treatment. Error bars represent 95% confidence intervals.

The monthly mortality rates of wild free-flying *An gambiae* s.l. which entered the experimental huts over the 12-month trial are presented in Fig 5 for both cement and mud wall huts. Wild mosquito mortality with Actellic® 300CS was >80% for the first 3–4 months of the trial and remained >50% for 6 months on both cement and mud-walled huts. Mortality was more persistent with Pirikool® 300CS; mortality was >80% for 6 months on both substrates and remained >50% for 10 months on cement walls and 11 months on mud walls.

### WHO cone bioassay residual activity on hut walls

**Fig 6:**
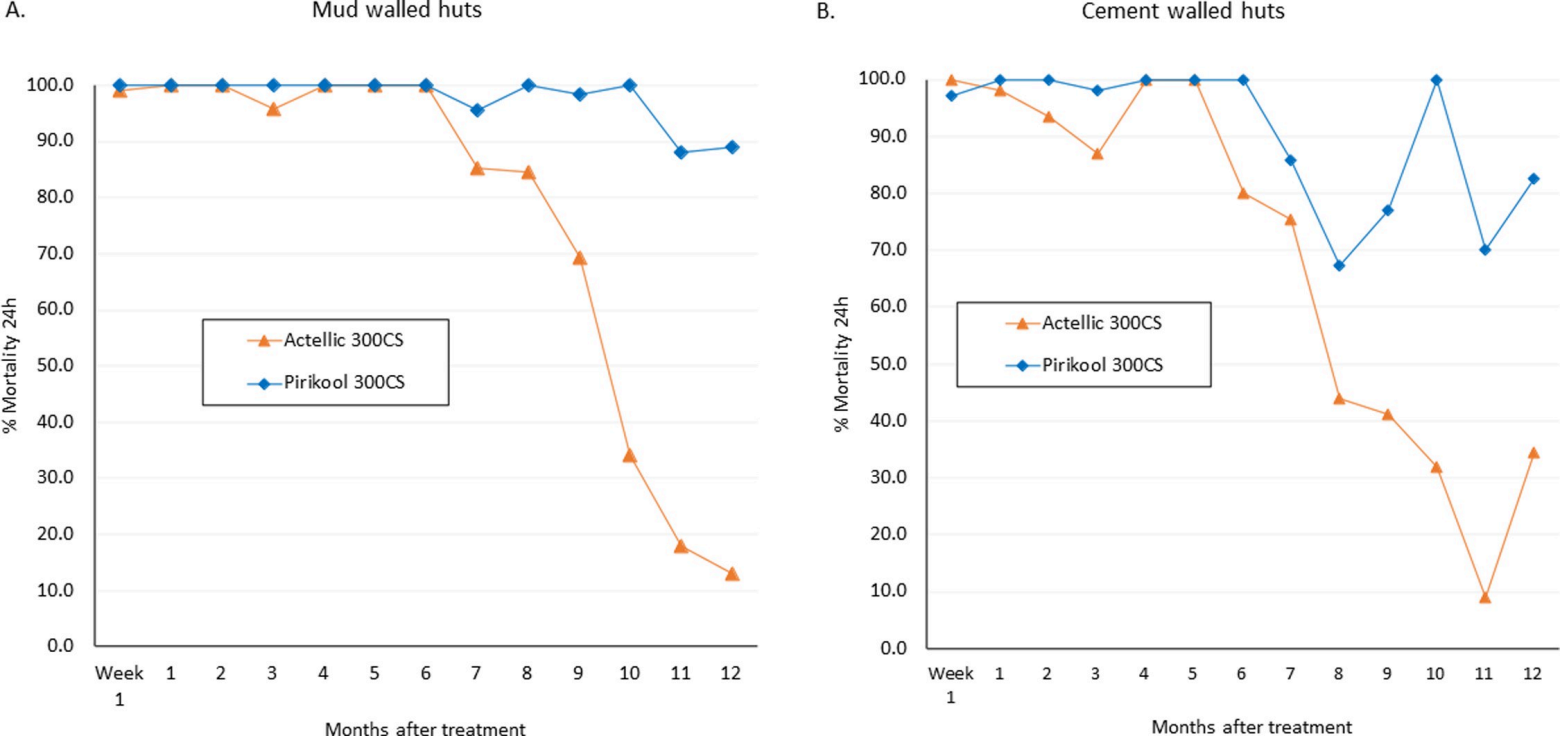
Residual efficacy of IRS treatments on cement–walled and mud-walled experimental huts: Cone bioassay mortality with laboratory susceptible *An*. *gambiae* Kisumu. Fifty (50) unfed 2–5 days old mosquitoes were tested in each hut per time point. Mortality in the control hut was <5% throughout.

**Fig 7:**
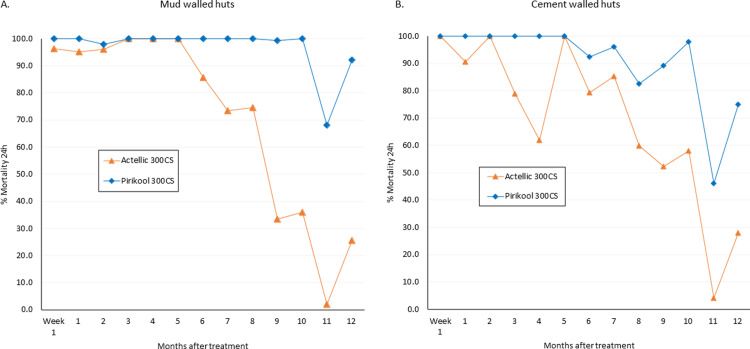
Residual efficacy of IRS treatments on cement–walled and mud-walled experimental huts: Cone bioassay mortality with pyrethroid-resistant *An*. *gambiae* s.l. Covè. Fifty (50) unfed 2–5 days old mosquitoes were tested in each hut per time point. Mortality in the control hut was <5% throughout.

The results from cone bioassays performed on the treated experimental hut wall surfaces during the trial using susceptible *An*. *gambiae* Kisumu strain and pyrethroid-resistant *An*. *gambiae* s.l. (Covè) are presented in Figs 6 and 7 respectively. Cone bioassay mortality with the susceptible Kisumu strain on Pirikool® 300CS treated hut walls was >80% for 10 months on cement walls and 12 months on mud walls (Fig 6). Similar results were obtained with the pyrethroid-resistant Cove strain (Fig 7). Cone bioassay mortality on Actellic® 300CS treated hut walls was also prolonged albeit to a lesser extent compared to Pirikool® 300CS; mortality of the susceptible Kisumu strain was >80% for 6 months on cement walls and 9 months on mud walls (Fig 6) while mortality with the pyrethroid-resistant strain was >80% mortality for 7 months on both mud and cement wall substrates (Fig 7).

### Quality of spray application

The summary results from the chemical analysis of filter papers attached to experimental hut walls during the spray applications with Pirikool®300CS and Actellic®300CS are presented in Table 4. The results showed that applications rates were generally within the 50% deviation limit from the target dose for Pirikool®300CS IRS as recommended by WHO [23] but were up to 61% to 74% deviation for Actellic® 300CS.

**Table 4:** Results from chemical analysis of filter papers from experimental huts treated with Pirikool® 300CS and Actellic® 300CS.

Hut treatments	Actellic Cement		Actellic Mud		Pirikool Cement		Pirikool Mud	
Replicate	**1**	**2**	**1**	**2**	**1**	**2**	**1**	**2**
Target dose (mg/m²)	1000	1000	1000	1000	1000	1000	1000	1000
Filter paper (mg/m²)	262	303	349	391	550	663	645	500
Deviation from target dose	-74	-69	-65	-61	-45	-34	-36	-49

## Discussion

To help increase the diversity of the portfolio of IRS insecticides and offer suitable options to malaria vector control programmes, new insecticides and new formulations of existing insecticides need to be developed [24]. This study demonstrated the efficacy of Pirikool® 300CS, a new capsule suspension formulation of pirimiphos-methyl (an organophosphate insecticide), for indoor residual spraying against malaria vector mosquitoes, in both laboratory and experimental hut studies. Applied in experimental huts with walls lined with local cement and mud substrates, Pirikool® 300CS showed a remarkable performance killing >85% of wild free-flying pyrethroid-resistant *Anopheles gambiae* s.l. that entered the experimental huts over 12 months.

Indoor residual spraying though an effective malaria control intervention, is logistically challenging as its implementation is labour intensive and requires political will and community engagement. As a result, longer-lasting IRS insecticide formulations which show prolonged activity necessitating only one campaign round to provide continuous control of pyrethroid-resistant malaria vectors in areas with extended malaria transmission, are in high demand. In a recent public consultation for preferred product characteristics for IRS products, WHO expressed the need for new IRS products with a residual efficacy on local wall substrates of at least 3 months and a preference for products lasting up to 12 months or more [25]. Residual efficacy of an IRS insecticide formulation is indicated by the number of months for which mortality in cone bioassays performed on treated local block substrates or home walls, remains over 80% [21]. Pirikool® 300CS demonstrated high and prolonged efficacy in both laboratory and experimental hut studies. In laboratory bioassays, the insecticide was efficacious throughout the 6-month study on mud and wood block substrates but lasted only 2–3 months on cement block substrates. Nevertheless, in the experimental hut trial, Pirikool® 300CS applied on both mud and cement walls induced high and prolonged vector mosquito mortality against wild free-flying pyrethroid-resistant malaria vectors and lasted for 10–12 months in monthly cone bioassays performed on treated hut walls. This will suggest a lack of correlation between the efficacy of Pirikool® 300CS on cement substrates in laboratory bioassays compared to experimental hut studies demonstrating longer residual efficacy with the latter compared to the former. This lack of correlation between laboratory cone bioassays and experimental hut in situ wall cone bioassays on cement substrates was also observed in studies involving another IRS insecticide under development [26]. Although the cement block substrates and cement wall plaster of the experimental huts were prepared using the same cement-sand ratio, there may be some differences in porosity or texture which could have contributed to the differences in residual efficacy of Pirikool® 300CS in both studies. Apart from the nature of the substrate, the durability of the bioefficacy of insecticides against malaria vectors on sprayable surfaces may also depend on environmental conditions [27, 28]. Reduced susceptibility of *Anopheles* vectors to organophosphates has been reported in WHO cylinder bioassays conducted at lower exposure temperatures (<26C) [27]. While temperature and humidity are held constant in laboratory bioassays, experimental hut conditions depend on the local natural environment which could vary considerably throughout the study. Differences in daily room temperature between the huts and laboratory assays may therefore have contributed to the difference in residual efficacy of Pirikool® 300CS on cement substrates in both studies. Nevertheless, compared to laboratory bioassays, experimental hut trials simulate household settings and thus provide a more realistic evaluation of the efficacy of an indoor intervention against wild vector mosquitoes. Transmission dynamics mathematical models have also indicated their potential to predict vector control product performance against clinical malaria when applied in communities [29]. Considering the investments that go into the development of new vector control products, it is advisable to consider experimental hut trials in addition to laboratory bioassays when investigating their efficacy for product registration. As shown in this study, limiting efficacy studies for product registration to laboratory bioassays could provide misguided information about the duration of the residual efficacy of new IRS products.

Overall wild vector mosquito mortality and residual efficacy in the experimental huts were higher with Pirikool® 300CS compared to Actellic® 300CS. This could be attributed to a lower application rate of Actellic® 300CS compared to Pirikool® 3000CS on the hut walls as indicated by the chemical analysis results. It was thus difficult to make a reliable direct comparison between Actellic® 300CS and Pirikool® 300CS from these results which may constitute a limitation of the study. Nevertheless, even at a sub-standard application rate, Actellic® 300CS killed 66–70% of wild vector mosquitoes entering the experimental huts over 12 months and showed a cone bioassay residual efficacy duration of up to 6–9 months which re-iterates the enhanced performance of micro-encapsulated pirimiphos-methyl formulations for IRS. This duration is beyond the expected duration of residual efficacy recommended for Actellic® 300CS by WHO (4–6 months) [10]. It also exceeds the duration of residual efficacy of Actellic® 300CS reported in more recent experimental huts studies conducted against the same vector population [30] where filter paper analysis showed higher application rates. This will suggest a poor correlation between the efficacy of the organophosphate IRS insecticide formulation in experimental huts and the application rates indicated by filter paper analysis results; huts which showed filter paper application rates as low as 26% to 41% of the target dose in our study, performed within expectations. Further studies to better understand the relevance of filter paper analysis in experimental hut trials and community evaluations of IRS products will be useful.

The main purpose of this study was to assess the performance of Pirikool® 300CS to determine whether the insecticide was efficacious for indoor residual spraying as part of a dossier preparation for product registration with the WHO PQT/VC. The results showed a high insecticidal effect against wild pyrethroid-resistant malaria vectors and prolonged residual efficacy on local wall substrates lasting 10–12 months which exceeds WHO’s minimum expectations of 3 months. The residual effect of Pirikool® 300CS against pyrethroid-resistant vector mosquitoes determined in this study is much longer than what has been reported with most pyrethroid and carbamate insecticides available on the WHO PQT/VC list [31]. The insecticide also showed comparable residual efficacy to other newly developed clothianidin based IRS insecticides which lasted 10–12 months when applied on mud and cement wall substrates in similar experimental hut trials and household studies in Benin [21, 32, 33]. Pirikool® 300CS, therefore, shows the potential to provide significant control of malaria transmitted by insecticide-resistant vector mosquitoes when deployed for IRS on a large scale.

## Conclusion

Pirikool® 300CS induced high mortality against wild free-flying pyrethroid-resistant *An gambiae* s.l. (85%) over 12 months in experimental huts in Benin. The insecticide also provided prolonged residual vector control which lasted for 10–12 months on both mud and cement experimental hut wall substrates. IRS with Pirikool® 300CS, therefore, has the potential to provide significant control of malaria transmitted by pyrethroid-resistant malaria vectors. Its addition to the current portfolio of IRS insecticides will provide an extra choice of microencapsulated pirimiphos-methyl for IRS.

## Supporting information

S1 Table Detailed experimental hut data against wild free-flying pyrethroid resistant *An gambiae* s.l. in Cove, Benin. (XLSX)

## References

[1] WHO. Guidelines for malaria vector control. Geneva, Switzerland: World Health Organization. 2019. 30844152

[2] Pluess B, Tanser FC, Lengeler C, Sharp BL. Indoor residual spraying for preventing malaria. The Cochrane database of systematic reviews. 2010;2010(4):CD006657–CD. doi: 10.1002/14651858.CD006657.pub2 . 20393950 PMC6532743

[3] WHO. World Malaria Report 2020. Geneva, Switzerland: World Health Organisation. 2020.

[4] Tangena J-AA, Hendriks CMJ, Devine M, Tammaro M, Trett AE, Williams I, et al. Indoor residual spraying for malaria control in sub-Saharan Africa 1997 to 2017: an adjusted retrospective analysis. Malaria Journal. 2020;19(1):150. doi: 10.1186/s12936-020-03216-6 32276585 PMC7149868

[5] Oxborough RM. Trends in US President’s Malaria Initiative-funded indoor residual spray coverage and insecticide choice in sub-Saharan Africa (2008–2015): urgent need for affordable, long-lasting insecticides. Malar J. 2016;15:146. Epub 2016/03/10. doi: 10.1186/s12936-016-1201-1 ; PubMed Central PMCID: PMC4784374. 26957210 PMC4784374

[6] Yeebiyo Y, Dengela D, Tesfaye AG, Anshebo GY, Kolyada L, Wirtz R, et al. Short persistence of bendiocarb sprayed on pervious walls and its implication for the indoor residual spray program in Ethiopia. Parasit Vectors. 2016;9:266. Epub 2016/05/07. doi: 10.1186/s13071-016-1549-7 ; PubMed Central PMCID: PMC4858853. 27151229 PMC4858853

[7] Fuseini G, Ebsworth P, Jones S, Knight D. The efficacy of ACTELLIC 50 EC, pirimiphos methyl, for indoor residual spraying in Ahafo, Ghana: area of high vector resistance to pyrethroids and organochlorines. J Med Entomol. 2011;48(2):437–40. Epub 2011/04/13. doi: 10.1603/me09286 . 21485386

[8] Oxborough RM, Kitau J, Jones R, Feston E, Matowo J, Mosha FW, et al. Long-lasting control of Anopheles arabiensis by a single spray application of micro-encapsulated pirimiphos-methyl (Actellic® 300 CS). Malaria journal. 2014;13(1):1–16. 24476070 10.1186/1475-2875-13-37 PMC3914366

[9] Rowland M, Boko P, Odjo A, Asidi A, Akogbeto M, N’Guessan R. A new long-lasting indoor residual formulation of the organophosphate insecticide pirimiphos methyl for prolonged control of pyrethroid-resistant mosquitoes: an experimental hut trial in Benin. PloS one. 2013;8(7):e69516. doi: 10.1371/journal.pone.0069516 23936033 PMC3720653

[10] WHO. Report of the sixteenth WHOPES working group meeting: WHO/HQ, Geneva, 22–30 July 2013: review of Pirimiphos-methyl 300 CS, Chlorfenapyr 240 SC, Deltamethrin 62.5 SC-PE, Duranet LN, Netprotect LN, Yahe LN, Spinosad 83.3 Monolayer DT, Spinosad 25 Extended release GR. World Health Organisation. 2013;https://apps.who.int/iris/bitstream/handle/10665/90976/9789241506304_eng.pdf?sequence=1.

[11] Chaccour C, Zulliger R, Wagman J, Casellas A, Nacima A, Elobolobo E, et al. Incremental impact on malaria incidence following indoor residual spraying in a highly endemic area with high standard ITN access in Mozambique: results from a cluster‐randomized study. Malaria journal. 2021;20(1):1–15. doi: 10.1186/s12936-020-03550-9 33568137 PMC7877039

[12] Wagman JM, Varela K, Zulliger R, Saifodine A, Muthoni R, Magesa S, et al. Reduced exposure to malaria vectors following indoor residual spraying of pirimiphos-methyl in a high-burden district of rural Mozambique with high ownership of long-lasting insecticidal nets: entomological surveillance results from a cluster-randomized trial. Malaria Journal. 2021;20(1):1–17. doi: 10.1186/s12936-020-03550-9 33478533 PMC7819201

[13] Abong’o B, Gimnig JE, Torr SJ, Longman B, Omoke D, Muchoki M, et al. Impact of indoor residual spraying with pirimiphos-methyl (Actellic 300CS) on entomological indicators of transmission and malaria case burden in Migori County, western Kenya. Scientific reports. 2020;10(1):1–14. doi: 10.1038/s41598-019-56847-4 32161302 PMC7066154

[14] Gogue C, Wagman J, Tynuv K, Saibu A, Yihdego Y, Malm K, et al. An observational analysis of the impact of indoor residual spraying in Northern, Upper East, and Upper West Regions of Ghana: 2014 through 2017. Malaria Journal. 2020;19(1):1–13. doi: 10.1186/s12936-019-3075-5 32652994 PMC7353711

[15] Wagman J, Cissé I, Kone D, Fomba S, Eckert E, Mihigo J, et al. Rapid reduction of malaria transmission following the introduction of indoor residual spraying in previously unsprayed districts: an observational analysis of Mopti Region, Mali, in 2017. Malaria Journal. 2020;19(1):1–10. doi: 10.1186/s12936-019-3075-5 32950056 PMC7501620

[16] Salako AS, Dagnon F, Sovi A, Padonou GG, Aïkpon R, Ahogni I, et al. Efficacy of Actellic 300 CS-based indoor residual spraying on key entomological indicators of malaria transmission in Alibori and Donga, two regions of northern Benin. Parasites & vectors. 2019;12(1):1–14. doi: 10.1186/s13071-019-3865-1 31888730 PMC6937814

[17] Protopopoff N, Mosha JF, Lukole E, Charlwood JD, Wright A, Mwalimu CD, et al. Effectiveness of a long-lasting piperonyl butoxide-treated insecticidal net and indoor residual spray interventions, separately and together, against malaria transmitted by pyrethroid-resistant mosquitoes: a cluster, randomised controlled, two-by-two factorial design trial. Lancet. 2018;391(10130):1577–88. Epub 2018/04/16. doi: 10.1016/S0140-6736(18)30427-6 ; PubMed Central PMCID: PMC5910376. 29655496 PMC5910376

[18] Wagman J, Cissé I, Kone D, Fomba S, Eckert E, Mihigo J, et al. Combining next-generation indoor residual spraying and drug-based malaria control strategies: observational evidence of a combined effect in Mali. Malaria Journal. 2020;19(1):1–11. doi: 10.1186/s12936-020-03361-y 32799873 PMC7429948

[19] WHO. Norms, standards and processes underpinning WHO vector control policy recommendations. 2020.

[20] Agossa FR, Padonou GG, Koukpo CZ, Zola-Sahossi J, Azondekon R, Akuoko OK, et al. Efficacy of a novel mode of action of an indoor residual spraying product, SumiShield(R) 50WG against susceptible and resistant populations of Anopheles gambiae (s.l.) in Benin, West Africa. Parasit Vectors. 2018;11(1):293. Epub 2018/05/12. doi: 10.1186/s13071-018-2869-6 ; PubMed Central PMCID: PMC5946391. 29747684 PMC5946391

[21] WHO. Guidelines for testing mosquito adulticides for indoor residual spraying and treatment of mosquito nets. World Health Organisation. 2006;Geneva, Switzerland.

[22] Ngufor C, N’Guessan R, Fagbohoun J, Subramaniam K, Odjo A, Fongnikin A, et al. Insecticide resistance profile of Anopheles gambiae from a phase II field station in Cove, southern Benin: implications for the evaluation of novel vector control products. Malar J. 2015;14:464. Epub 2015/11/20. doi: 10.1186/s12936-015-0981-z ; PubMed Central PMCID: PMC4652434. 26581678 PMC4652434

[23] WHO. Data requirements and protocol for determining non-inferiority of insecticide-treated net and indoor residual spraying products within an established WHO intervention class. Geneva, Switzerland. 2018;https://apps.who.int/iris/bitstream/handle/10665/276039/WHO-CDS-GMP-2018.22-eng.pdf?ua=1.

[24] WHO. Global Technical Strategy for malaria 2016–2030. Geneva, Switzerland: World Health Organization. 2015.

[25] WHO. Preferred product characteristics: indoor residual spraying products for malaria transmission control in areas with insecticide-resistant mosquito populations; Draft for public consultation. World Health Organisation. 2021;https://cdn.who.int/media/docs/default-source/malaria/ppcs-etc/who-ucn-gmp-2021-02-eng.pdf?sfvrsn=dbd89f86_10.

[26] Ngufor C, Govoetchan R, Fongnikin A, Vigninou E, Syme T, Akogbeto M, et al. Efficacy of broflanilide (VECTRON™ T500), a new meta-diamide insecticide, for indoor residual spraying against pyrethroid-resistant malaria vectors. bioRxiv. 2020:2020.11.06.367961. doi: 10.1101/2020.11.06.367961 PMC8042056 33846394

[27] Glunt KD, Paaijmans KP, Read AF, Thomas MB. Environmental temperatures significantly change the impact of insecticides measured using WHOPES protocols. Malaria Journal. 2014;13(1):350. doi: 10.1186/1475-2875-13-350 25187231 PMC4162960

[28] Dengela D, Seyoum A, Lucas B, Johns B, George K, Belemvire A, et al. Multi-country assessment of residual bio-efficacy of insecticides used for indoor residual spraying in malaria control on different surface types: results from program monitoring in 17 PMI/USAID-supported IRS countries. Parasit Vectors. 2018;11(1):71. Epub 2018/02/01. doi: 10.1186/s13071-017-2608-4 ; PubMed Central PMCID: PMC5791726. 29382388 PMC5791726

[29] Sherrard-Smith E, Griffin JT, Winskill P, Corbel V, Pennetier C, Djenontin A, et al. Systematic review of indoor residual spray efficacy and effectiveness against Plasmodium falciparum in Africa. Nat Commun. 2018;9(1):4982. Epub 2018/11/28. doi: 10.1038/s41467-018-07357-w ; PubMed Central PMCID: PMC6255894. 30478327 PMC6255894

[30] Ngufor C, Govoetchan R, Fongnikin A, Vigninou E, Syme T, Akogbeto M, et al. Efficacy of broflanilide (VECTRON T500), a new meta-diamide insecticide, for indoor residual spraying against pyrethroid-resistant malaria vectors. Sci Rep. 2021;11(1):7976. Epub 2021/04/14. doi: 10.1038/s41598-021-86935-3 ; PubMed Central PMCID: PMC8042056. 33846394 PMC8042056

[31] WHO. List of WHO prequalified vector control products. Geneva, Switzerland: World Health Organization. 2021;https://www.who.int/pq-vector-control/prequalified-lists/PrequalifiedProducts27January2020.pdf?ua=1.

[32] Fongnikin A, Houeto N, Agbevo A, Odjo A, Syme T, N’Guessan R, et al. Efficacy of Fludora® Fusion (a mixture of deltamethrin and clothianidin) for indoor residual spraying against pyrethroid-resistant malaria vectors: laboratory and experimental hut evaluation. Parasites & Vectors. 2020;13(1):466. doi: 10.1186/s13071-020-04341-6 32917255 PMC7488472

[33] Agossa FR, Padonou GG, Fassinou A, Odjo EM, Akuoko OK, Salako A, et al. Small-scale field evaluation of the efficacy and residual effect of Fludora((R)) Fusion (mixture of clothianidin and deltamethrin) against susceptible and resistant Anopheles gambiae populations from Benin, West Africa. Malar J. 2018;17(1):484. Epub 2018/12/31. doi: 10.1186/s12936-018-2633-6 ; PubMed Central PMCID: PMC6311023. 30594207 PMC6311023

